# Speech perception in modulated noise assessed in bimodal CI users

**DOI:** 10.1007/s00106-023-01321-x

**Published:** 2023-08-08

**Authors:** Tobias Weißgerber, Timo Stöver, Uwe Baumann

**Affiliations:** 1 Audiological Acoustics, Department of Otolaryngology, University Hospital Frankfurt, Goethe University Frankfurt, Theodor-Stern-Kai 7, 60590 Frankfurt am Main, Germany; 2 Department of Otorhinolaryngology, University Hospital Frankfurt, Goethe University Frankfurt, Frankfurt am Main, Germany

**Keywords:** Speech audiometry, Hearing loss, Auditory implants, Hearing disorders, Hearing aids

## Abstract

**Background:**

Although good speech perception in quiet is achievable with cochlear implants (CIs), speech perception in noise is severely impaired compared to normal hearing (NH). In the case of a bimodal CI fitting with a hearing aid (HA) in the opposite ear, the amount of residual acoustic hearing influences speech perception in noise.

**Objective:**

The aim of this work was to investigate speech perception in noise in a group of bimodal CI users and compare the results to age-matched HA users and people without subjective hearing loss, as well as with a young NH group.

**Materials and methods:**

Study participants comprised 19 bimodal CI users, 39 HA users, and 40 subjectively NH subjects in the age group 60–90 years and 14 young NH subjects. Speech reception thresholds (SRTs) in noise were adaptively measured using the Oldenburg Sentence Test for the two spatial test conditions S0N0 (speech and noise from the front) and multisource-noise field (MSNF; speech from the front, four spatially distributed noise sources) in continuous noise of the Oldenburg Sentence Test (Ol-noise) and amplitude-modulated Fastl noise (Fastl-noise).

**Results:**

With increasing hearing loss, the median SRT worsened significantly in all conditions. In test condition S0N0, the SRT of the CI group was 5.6 dB worse in Ol-noise than in the young NH group (mean age 26.4 years) and 22.5 dB worse in Fastl-noise; in MSNF, the differences were 6.6 dB (Ol-noise) and 17.3 dB (Fastl-noise), respectively. In the young NH group, median SRT in condition S0N0 improved by 11 dB due to gap listening; in the older NH group, SRTs improved by only 3.1 dB. In the HA and bimodal CI groups there was no gap listening effect and SRTs in Fastl-noise were even worse than in Ol-noise.

**Conclusion:**

With increasing hearing loss, speech perception in modulated noise is even more impaired than in continuous noise.

## Background and aim

For people with severe sensorineural hearing loss who cannot be adequately treated with conventional hearing aids (HAs), fitting a cochlear implant (CI) is the therapy of choice. Because often both ears are affected by hearing disorders, an HA is commonly used in the opposite ear (bimodal fitting) if the HA fitting is still successful. Otherwise, if the prerequisites for CI indication are met (i.e., speech perception of ≤ 60% with HA, measured with the Freiburg monosyllabic speech test [11] at 65 dB SPL in free-field, according to the current guideline on CI fitting [1]), a CI fitting is also performed in the second ear (bilateral fitting). Under quiet listening conditions, good speech perception is achieved in most CI users. However, in everyday listening environments, speech is often superposed by background noise. It is known that in such situations the speech perception of CI users is significantly impaired compared to normal-hearing (NH) people [18, 19]. The healthy auditory system is able to filter information in noisy situations by evaluating the signal differences of both ears and to enhance the perception of desired signals (cocktail party effect; [5]). Here, among other things, the spatial separation of the noise sources is exploited. Furthermore, everyday background noise often shows short temporal pauses or gaps, which for a short time improve the signal-to-noise ratio and increase the perception of speech in NH people (“gap listening,” “glimpsing” [6]). Rader and coauthors were unable to demonstrate the ability of gap listening in bilateral CI users and bimodal CI users with electric-acoustic stimulation (EAS) when compared with NH users; [19]. In the aforementioned study, the NH participants were younger on average than the CI groups. The results published by Füllgrabe [10] show that temporal processing decreases with age, even in the absence of peripheral hearing loss. Sensitivity to temporal fine structure decreased with age in both monaural and binaural psychoacoustical experiments, starting in early midlife.

The aim of the present study was to investigate speech perception in noise in different listening environments in CI users with bimodal fitting and to compare the results with groups of HA users and subjectively NH users of the same age. In particular, the influence of the contralateral ear with HA on spatial release from masking (SRM) and gap listening was investigated.

## Material and methods

### Participants

**Fig. 1:**
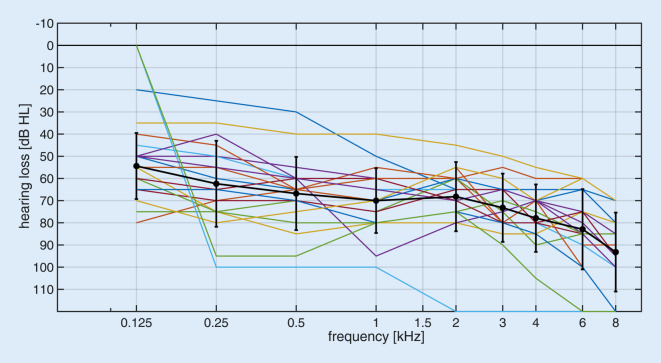
Pure-tone audiograms of the contralateral ear (hearing aid side) of the 19 participants and mean audiogram with standard deviation (*black*)

**Fig. 2:**
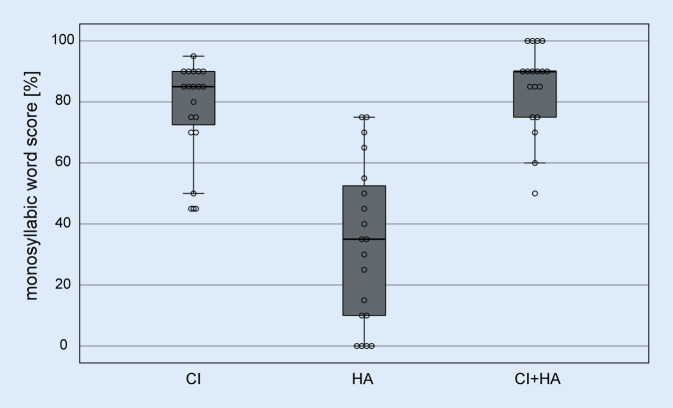
Boxplots and individual data of monosyllabic word score with CI, HA, and binaural with CI and HA (*n* = 19). Measurements were taken in free-field at a sound pressure level of 65 dB. *CI* cochlear implant, *HA* hearing aid

The present study included a total of 19 participants (14 male, 5 female) with an age of at least 60 years. Clear signs of dementia were excluded using a screening test (at least 9 points in the DemTect test; [14]). The age range of the participants was between 61.2 and 84.0 years (mean age ± standard deviation: 70.7 ± 6.2 years) and all of them were native German speakers. All participants were unilaterally fitted with a CI (Cochlear, Macquarie, Australia) and used an HA in the opposite ear. All implants were of the type CI24RE (CA) or CI422. The speech processors used were either of type CP810 or CP910. All tests used a standard microphone directionality (sub-cardioid) and dynamic processing (i.e., ASC/ADRO if applicable) as used in the participant’s everyday program. Additional noise reduction (SNR-NR) in the CP910 processor was always disabled. The individual pure-tone audiograms and the pure-tone audiogram of the contralateral ear averaged over all participants are shown in Fig. 1. The mean hearing loss (averaged over the frequencies 500, 1000, 2000, and 4000 Hz) was 70.3 ± 14.2 dB HL. Boxplots of speech perception in quiet (Freiburg monosyllabic speech test [11] at 65 dB SPL in free-field) are shown in Fig. 2 for each ear separately and for binaural measurement. When measuring the ear with CI, the opposite ear was blocked with an earplug and additionally with an ear-enclosing earmuff. Mean monosyllabic word score in the ear fitted with a CI was 77.9 ± 15.6%, in the HA ear it was 33.4 ± 26.6%, and in the binaural measurement it was 84.7 ± 14.0%.

A group of 39 HA users (3 with HA right, 3 with HA left, 33 with HA bilateral; mean age: 76.0 ± 4.7 years, further demographic data shown in [15]), a group of 40 people without subjective hearing loss (mean age: 69.3 ± 7.1 years), and a young group of NH people (*n* = 14, mean age: 26.4 ± 5.4 years) served as comparison groups. The results for speech perception in noise of the groups without subjective hearing loss and young NH persons are taken from the publication by Weißgerber et al. [25].

The study was approved by the Ethics Committee of the Department of Medicine of the Johann Wolfgang Goethe University in Frankfurt am Main under reference number 164/13.

### Speech perception in noise

The measurements were made in an anechoic room with dimensions of 4.1 m × 2.6 m × 2.1 m (length × width × height). The reproduction system consisted of 128 independent loudspeaker channels, which were arranged in a rectangular array in the horizontal plane at a height of 1.20 m. By using the reproduction method of wave field synthesis [2], it is possible to create virtual sound sources in almost any position inside or outside the room. More detailed information on the playback system is available from Weißgerber [24].

With the Oldenburg Sentence Test [21–23], the speech reception threshold (SRT) was determined for 50% speech intelligibility in noise. The noise level was kept constant at a sound pressure level of 65 dB, the speech level was determined according to the method of Brand and Kollmeier [3] according to the number of correctly recognized words.

The test was conducted in closed-set mode, i.e., the participant was alone in the test room and, after hearing the sentence, had to select the elements of the sentence on a touchscreen by touching the corresponding words on the touchscreen that he or she had understood. Prior to the start of the study tests, each participant was given a training run in quiet at a fixed speech level of 65 dB SPL and an additional adaptive test list (both test lists with 30 items each) in noise. This was followed by four runs of the Oldenburg Sentence Test with 20-item test lists in random order. Two different spatial configurations of speech and noise with two different noise types were examined.

The two noises were, on the one hand, the temporally continuous Oldenburg noise (Olnoise), whose long-term spectrum matches that of the Oldenburg Sentence Test word material [21]. On the other hand, the amplitude-modulated, speech-simulating fluctuating noise according to Fastl (Fastl-Noise; [8]) was used. The spectral distribution of the amplitude modulation reaches a maximum at a modulation frequency of 4 Hz, which corresponds approximately to the average number of spoken syllables per second in Western speech [9].

The two spatial test configurations were S0N0 and the “multisource noise field” (MSNF) according to Rader et al. [19]. In S0N0, speech signal (S) and noise (N) were presented from the same direction of 0° frontally at a distance of 1.75 m from the participant. For this purpose, four adjacent loudspeakers of the playback system were used to obtain a sufficient sound pressure level at the position of the participant [26].

In the MSNF test condition, the speech signal was also generated from the front loudspeakers at 0° position with a distance of 1.75 m from the participant. By wave field synthesis, a diffuse noise field was created with four virtual noise sources at the ±28.6 and ±151.4° positions with a distance of 1.25 m from the center of the participant’s head [26]. The four virtual noise sources reproduced the noise in a temporally decorrelated manner. The MSNF loudspeaker configuration was chosen to simulate everyday conversational situations in a noisy environment, such as conversations in a restaurant.

### Statistical analysis

The collected data and measured values were processed and analyzed using the statistical program SPSS 27 (IBM, Armonk, NY, USA). The target variables of the different tests were checked for normal distribution using the Shapiro–Wilk test. Since none of the target variables showed a normal distribution, further analysis was performed using nonparametric procedures only. To test for significant differences, the Mann–Whitney *U* test (test metric: *Z*_*u*_) was used for two independent samples and the Kruskal–Wallis test (test metric: *H*) was used for more than two independent samples. If there were two dependent samples, the Wilcoxon test (test metric: *Z*_*w*_) was applied. In the case of multiple pair comparisons, the significance level was corrected (Benjamini–Hochberg procedure).

## Results

**Fig. 3:**
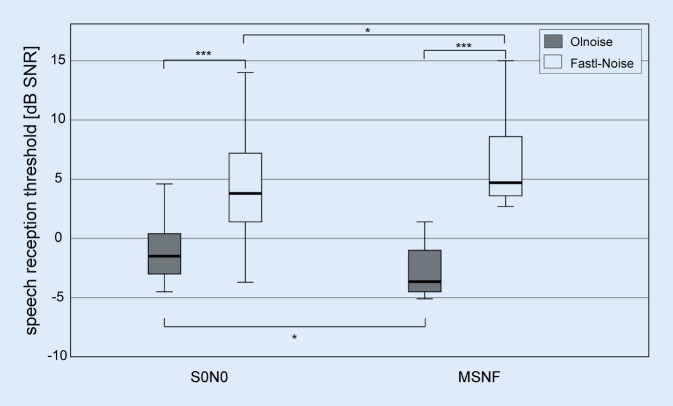
Boxplots of speech reception thresholds for the spatial test conditions S0N0 and MSNF and the two noises Olnoise (*gray boxes*) and Fastl-Noise (*white boxes*) for the 19 bimodal CI users. Lower thresholds in the SRT mean better speech perception (***p < 0.001, *p < 0.05). *Olnoise* temporally continuous Oldenburg noise, *Fastl-Noise* speech-simulating fluctuating noise according to Fastl. *MSNF* multisource noise field

Figure 3 shows the results of the bimodal CI group for the two test conditions S0N0 and MSNF and for the noise conditions Olnoise and Fastl-Noise, respectively. In the S0N0 test condition, the median SRT in the noise condition Olnoise was −1.5 dB SNR. In Fastl-Noise, an SRT of 4.5 dB SNR was achieved. The results in the Fastl-Noise were significantly worse (by 6 dB) than in the Olnoise condition (*Z*_*w*_ = −3.724, *p* < 0.001). In the MSNF test condition, a median SRT of −3.5 dB SNR was achieved in the Olnoise condition. In the Fastl-Noise condition, an SRT of 4.8 dB SNR was achieved. The results in Fastl-Noise were again significantly worse (8.3 dB, *Z*_*w*_ = −3.823, *p* < 0*.*001).

For the Olnoise, a significant impact of the spatial configuration (i.e., SRM) of 2 dB was found (*Z*_*w*_ = −2.593, *p* = 0.013). The deterioration of the SRT in the Fastl-Noise condition in the MSNF of 0.3 dB was also statistically significant (*Z*_*w*_ = −2.496, *p* = 0*.*013).

**Fig. 4:**
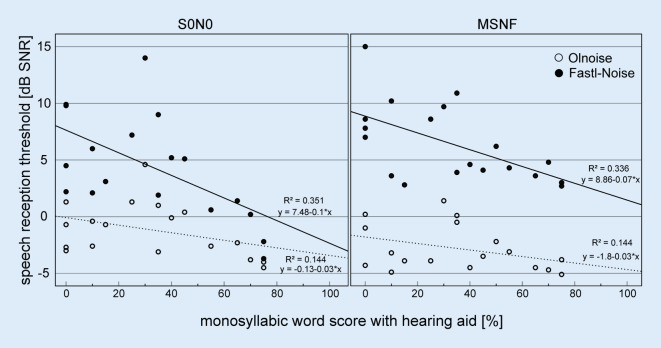
Scatterplots of the monosyllabic score (side of hearing aid) and speech reception threshold, respectively, for S0N0 (*left*) and MSNF (*right*) and the noise Fastl-Noise (*filled circles*) and Olnoise (*open circles*) conditions for the 19 bimodal cochlear implant users. In each case, the *dashed lines *show the linear regression line for Olnoise, and the *solid lines* for Fastl-Noise.* Olnoise* temporally continuous Oldenburg noise, *Fastl-Noise* speech-simulating fluctuating noise according to Fastl,* MSNF* multisource noise field

In the test condition S0N0, a significant correlation of the mean hearing loss of the HA-fitted side with the SRT was shown (Olnoise: ρ = 0.49, *p* = 0.037; Fastl-Noise: ρ = 0.40, *p* = 0.039). There was a significant negative correlation of SRTs in the Fastl-Noise condition with monosyllabic word score with HAs (see scatter plots in Fig. 4, S0N0: ρ = 0.61, *p* = 0.006; MSNF: ρ = 0.57, *p* = 0.012). No correlation between SRT and monosyllabic word score with CI was found.

**Fig. 5:**
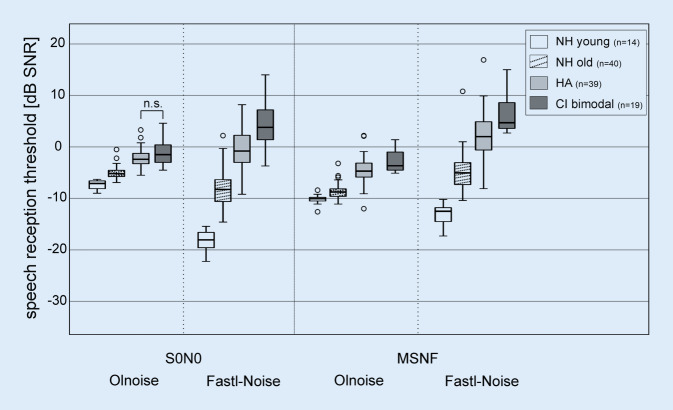
Boxplots of speech reception thresholds (SRTs) for the four study groups: young normal hearing (*NH*, *white*, *n* = 14), older NH (*shaded*, *n* = 40), hearing aid users (*HA*, *light gray*, *n* = 39), and bimodal CI users (*dark gray*, *n* = 19) in the spatial conditions S0N0 and MSNF and the two noise conditions Olnoise and Fastl-Noise. For each of the four test conditions, the SRTs of all groups differ significantly from each other, except for the pairwise comparison between the HA and CI groups in Olnoise and S0N0 (*n.* *s.* not significant).* Olnoise* temporally continuous Oldenburg noise, *Fastl-Noise* speech-simulating fluctuating noise according to Fastl,* MSNF* multisource noise field

The results of a comparison of the results of the bimodal CI group with groups of the same age with and without HAs and with a young NH group are shown in Fig. 5. For all test conditions, there was a significant effect of study group (S0N0 Olnoise: *H* = 72.911; S0N0 Fastl-Noise: *H* = 81.744; MSNF Olnoise: *H* = 74.054; MSNF Fastl-Noise: *H* = 80.475; all *df* = 3, all *p* < 0.001). Considering the group performance, those with more pronounced hearing loss had worse median SRTs than the groups with less or no hearing loss (i.e., NH young → NH old → HA users → bimodal CI). Only in the condition S0N0 using Olnoise, no difference in SRTs between the HA and CI group was found.

In the S0N0 test condition, the SRT of the CI group was 5.6 dB worse than in the young NH group in Olnoise and 22.5 dB worse in the Fastl-Noise condition. In MSNF, the differences were 6.6 dB (Olnoise) and 17.3 dB (Fastl-Noise), respectively. Compared with the HA group, SRTs were only 0.9 dB (S0N0) and 1.2 dB (MSNF) worse in the Olnoise condition, whereas in the Fastl-Noise condition the difference was 5.3 dB (S0N0) and 2.8 dB (MSNF).

In the young NH group, gap listening improved SRTs in the S0N0 condition by 11 dB (*Z*_*w*_ = −3.297, *p* < 0.001), in the older NH group by only 3.1 dB (*Z*_*w*_ = −5.216, *p* < 0.001). In the HA group, the Fastl-Noise condition resulted in a deterioration of SRTs compared to the Olnoise condition (1.6 dB, *Z*_*w*_ = −2.916, *p* = 0.004) as in the bimodal CI group.

In the young NH group, gap listening improved SRTs in the MSNF condition by 2.4 dB (*Z*_*w*_ = −3.297, *p* < 0.001). In the older NH group and in the group with HA fitting, the Fastl-Noise condition deteriorated SRTs compared to the Olnoise condition (NH old: 3.7 dB, *Z*_*w*_ = −5.276, *p* < 0.001; HA: 6.7 dB, *Z*_*w*_ = −5.443, *p* < 0.001) as in the bimodal CI group.

All of the study groups showed approximately comparable SRM (difference in median SVS of S0N0 and MSNF) of between 2 and 3.6 dB in the Olnoise condition.

## Discussion

Speech reception thresholds in noise were compared for three groups of participants (NH, HA users, bimodal CI users) of the same age (older than 60 years) and for one young NH group for the two spatial test conditions S0N0 and MSNF in continuous Olnoise and in temporally modulated Fastl-Noise conditions. With increasing hearing loss, SRTs worsened in all test conditions. Whereas the SRTs of the HA and bimodal CI group differed only slightly in the continuous Olnoise condition, the SRT of the bimodal CI group in the modulated Fastl-Noise condition was significantly worse than in all other groups.

### Impact of background noise on speech perception (“gap listening”)

The effect of gap listening could be shown in the young NH group in both spatial noise conditions S0N0 and MSNF and was in the same order of magnitude (S0N0: 11 dB, MSNF: 2.4 dB) as that reported by Rader and coauthors [19]. In the older NH group (with age-related hearing loss), gap listening in the S0N0 condition with only 3.1 dB was already 7.9 dB worse than in the young NH group. In the HA group and the bimodal CI group there was no effect of gap listening at all. On the contrary, SRT in modulated noise was even worse than in continuous Olnoise. These results are in line with previous studies using the same noise type in bimodal EAS and bilateral CI, respectively [19], or in bilateral CI users [26]. Hey et al. [12] also reported gap listening in NH individuals in a modulated ICRA7 noise (6 superimposed speakers, i.e., less modulated than the Fastl noise), whereas in CI users aged 43–80 years, SRTs deteriorated by a mean of 4 dB. In another work by Hey et al. [13], severe deficits in gap listening abilities in CI users compared to NH individuals (more than 20 dB worse SRT) were reported. Zirn et al. [27] also found deteriorated SRTs in a group of CI users (unilateral, bimodal, and bilateral data pooled) younger than 65 years at S0N0 in Fastl-Noise condition. Only in the work of Weißgerber and coworkers [26] a small effect of gap listening was found in a bimodal CI group (mean age: 49.6 ± 19.1 years). Although this effect could not be confirmed in the present work, a negative correlation of speech perception in the ear using an HA with SRT in Fastl-Noise was found in the bimodal CI group, which was not present in the Olnoise condition. Thus, with increasing contralateral acoustic hearing ability, the possibility of gap listening increases in bimodal CI fitting.

In addition to the limited dynamic range in the CI and HA group and the relatively low-frequency resolution in CI systems, there are also deficits in frequency selection with increasing age and for mild hearing losses, leading to poorer separation of speech and sounds and making it more difficult or impossible to detect temporal gaps [16]. Duquesnoy describes that older individuals aged 75–88 years with presbycusis are less able to use the temporal gaps in fluctuating noise than NH individuals [7]. Peters and coauthors showed that both age and hearing loss have a significant effect on speech perception, with the effects being greatest in modulated noise [17]. In young adults with NH, the improvement in SRTs due to gap listening was 4–7 dB, whereas in older individuals with hearing loss, only a 1.5-dB improvement was found. The results of van Summers and Molis [20] suggest that audibility of signals is not the main factor limiting gap hearing in mild-to-moderate hearing loss. The reduced ability to exploit temporal fluctuations in the masker continues to be present in the majority of study participants even when the presentation level is increased by up to 30 dB. Rather, limitations in the processing of suprathreshold speech could be responsible for the reduced gap listening ability, e.g., a possible deterioration in temporal resolution. The results presented by Füllgrabe show that temporal processing decreases with age, even in the absence of peripheral hearing loss [10]. Sensitivity to temporal fine structure decreased with age in both monaural and binaural listening tests, starting in early midlife. Therefore, group comparisons of gap listening ability must always take into account the age of the different groups. In the present study, the ages of the three comparison groups without and with HA as well as with bimodal CI fitting were age-matched as closely as possible, and there were also no significant group differences in dementia screening.

### Impact of the spatial test condition (spatial release from masking)

The role of binaural processing is shown in the comparison of the test condition S0N0 with the MSNF. Spatial release from masking leads to improved SRTs compared to the S0N0 condition due to the head shadow effect and the binaural squelch effect [4]. In the study by Duquesnoy [7], SRM was found to be 5–9 dB for a noise signal from the side and speech signal from the front compared to S0N0 in young NH individuals and 3–4 dB in older individuals with age-related hearing loss.

In the present study, SRM in the MSNF condition with continuous Olnoise was comparable in all study groups (2–3.5 dB). The results in SRM of the bimodal CI group are consistent with previous results obtained in the same test setup with bimodal and bilateral CI users (approximately 2 dB SRM; [26]) and with bimodal EAS and bimodal CI, respectively, (approximately 3 dB SRM; [19]).

In the MSNF condition with Fastl-Noise, the effect of SRM cannot be assessed individually, because in addition to the difference in spatial configuration, the modulation properties (monaurally) of the noise (four uncorrelated superposed sources with Fastl-Noise in MSNF vs. one source of Fastl-Noise in S0N0) are different. Although the noise sources are spatially separated from the target speaker and additional temporal gaps are in the noise signals, in this test condition the worst SRTs compared to all other test conditions are achieved in all three older study groups, which could be due to a combined effect of both degraded binaural processing and temporal processing.

## Practical conclusion

- To better assess the hearing performance of people with hearing loss in everyday listening conditions, the use of temporally fluctuating noise in speech audiometry is recommended.
- Furthermore, a test condition with spatially separated noise should be used. Even with only mild hearing loss, gap listening ability decreases with age.
- In progressive hearing loss with hearing aid or cochlear implant fitting, speech perception is even more impaired in modulated noise than in continuous noise.
- In continuous noise, the ability of spatial unmasking (spatial release from masking) in all of the groups studied is present to a comparable degree.

## Declarations

All procedures performed in studies involving human participants or on human tissue were in accordance with the ethical standards of the institutional and/or national research committee and with the 1975 Helsinki declaration and its later amendments or comparable ethical standards. Informed consent was obtained from all individual participants included in the study.

The supplement containing this article is not sponsored by industry.
